# A Sensible Judge’s Charge to the Jury in a Suit for Malpractice

**Published:** 1861-05

**Authors:** 


### A SENSIBLE JUDGE'S CHARGE TO THE JURY IN A SUIT FOR MALPRACTICE.

So much ignorance and prejudice in regard to the real moral and legal responsibilities of the surgeon have been frequently evinced in the charge of judges in suits for malpractice, that one in which the real position of a surgeon is appreciated deserves to be spread before the profession.

The charge, which we copy, is in strong contrast with the position taken by Judge Bredin, of Pittsburg, who, in a suit for malpractice, which occurred some years ago, said, that the defendant "was bound to bring to his aid the skill necessary for a surgeon to set a leg, so as to make straight, and of equal length with the other when healed, and if he did not, was accountable in damage, just as a stone-mason would be in building a wall of poor materials, so that the wall would fall down."

It is now many years since an eminent surgeon of this city was persecuted in this manner, and obliged to pay damages to the amount of more than $3,000 on account simply of the failure of an operation for cataract on a charity patient.

The late Dr. Horner was, a short time previously to his death, sued for damages to the amount of *one hundred thousand dollars*, in a case where death happened to follow an important operation on the wife of a poor man. Dr. Henry H. Smith acted as an assistant at the operation, and although he had never before seen the patient, nor been consulted in reference to the operation, he was included in the prosecution, and Dr. Horner dying before the case was tried, Dr. Smith was compelled to stand his trial. Had not the bad character of the plaintiff been proved, and the malicious intent of the prosecution been made evident, the surgeon might, from the stupidity of a jury, have been made to suffer severely.

This action was brought by Daniel S. Hamilton against Drs. Squire, Wey, and Smith, for damages alleged to have been sustained by the plaintiff in consequence of a surgical operation performed upon his knee by the defendants. The operation consisted in the removal of a loose or floating cartilage from the knee-joint, by means of what is known among surgical writers as the *valvular mode of incision.* Inflammation of the joint ensued, its disorganization followed, and the ultimate result was a stiff knee; the limb being slightly flexed and bowed laterally, in consequence of destruction of the articular cartilages, and the expanded extremities of the bones entering into the composition of the joint, on its inner side. Damage was claimed to the amount of $5,000. After a protracted trial, the case was submitted to the jury, in the following charge by Judge Campbell. The jury failed to agree, standing one for plaintiff and eleven for the defendants.

*Gentlemen of the Jury:*—Every person who enters a learned profession, whether the law or surgery, undertakes to bring to it the exercise of a reasonable, fair, and competent degree of skill.

Invariable success does not attend professional men, any more than those engaged in other pursuits. Indeed, success must with them sometimes depend on other instrumentalities than mere skill. Courts and juries are fallible and may err, and the best advice and labor of counsel in the law may be in vain; and habits of life unknown, and hereditary diseases, and neglect of directions, and carelessness of nurses, may defeat the labors of the most skillful surgeon. Both the lawyer and surgeon, when they undertake professional business, agree to be responsible for the want of ordinary care—such care as ordinarily prudent men bestow upon their business. This is the responsibility which the law imposes upon them. But it is said the professional man is also bound to use his best judgment, and that judgment should be an enlightened one. This is true; but in cases where there is great difference of opinion among the most skillful and experienced as to surgery, where the most eminent men in the profession differ as to the methods of performing operations, the surgeon who possesses the necessary qualifications will not be held responsible for errors of judgment. He will be chargeable with error only when such error arises from want of reasonable, ordinary skill and diligence, especially if the general charac- of the operation and treatment has been honest and intelligent.

1st. Was this a proper operation under the circumstances of the case?

2d. Was it proper without the bandage or compression? *

3d. Was the valvular method a proper one?

4th. Was the place where the cartilage was taken out a proper one?

5th. Was the after-treatment proper?

To all these questions some of the most eminent physicians in the State, and I may say among the most eminent in the United States, have given you an affirmative answer. Others, on the part of the plaintiff, who may be equally intelligent, but who have not had equal experience, answer in the negative. Now, in such a case, where there is such difference of opinion, and certainly with the experienced men in the defendant's favor, they should not be held reliable for an error of judgment, even if you should be of the opinion that they did err.

The operation being thus, for the purposes of this suit, warrantable, and the method, place and treatment proper, was the operation performed, and the after-treatment continned, with reasonable skill and care—such skill and care as would be required at the hands of prudent, competent surgeons?

Now, the contract of a surgeon is not to warrant a cure, except such contract be expressly made. He contracts to

* Dr. March, Dr. Markoe, and Dr. French, the three surgeons who have operated for the removal of the loose cartilages, all unite in saying that the operation is warranted without resort to the bandage.

use his best skill, care, and attention. In this particular operation, it appears by the evidence of that eminent surgeon, Dr. March, that he had been uniformly successful. But taking the results of operations by other surgeons, so far as reported, one-fourth are *not* successful. It would not do, therefore, to hold up the responsibility of every surgeon in the land with that of one of the most eminent.

As to the manner in which the operation was performed, you have had the evidence of the defendants, together with that of Mr. Birchfield. If the delay in the operation was caused by the plaintiff, and therefore the time was protracted, the plaintiff cannot recover for any injury caused by such acts of his own.

As to the care and attention after the operation, as I understand, no complaint was made; but, on the contrary, the care and attention were constant, and such as might be expected of a kind and careful surgeon.

I have already observed that, from the evidence, it appears one-fourth of such operations are not successful. The want of success is not necessarily the want of skill.

Three-fourths of the cases are successful; and if the plaintiff had been among the successful number, if his limb had been entirely restored, he might, like the lame man healed by the Apostle, have "ran and leaped with joy." That it was not successful, is undoubtedly a great misfortune to him. Whether it was the fault of the defendants, is for you to say by your verdict.

You must take this case and determine it according to the evidence under your oaths.

In the case of Dr. Smith, it is claimed that he had nothing to do with the operation; that he was merely a looker-on, invited by Dr. Squire, as a simple act of courtesy; and that in point of fact he was not present until the operation was nearly completed, and when the chloroform was sent for. If you believe the evidence of the defendants on this point, of course you should render a verdict in his favor.

Then, if you find that this operation was not performed by Dr. Squires and Dr. Wey with ordinary skill, care, and diligence, you should find a verdict for the plaintiff.

On the other hand, if you find that they did perform the operation with ordinary skill and care, and such as would be required of surgeons holding a responsible position in their profession, then your verdict should be in favor of the defendants.—*M. & S. Reporter*.

